# The Emotional Burden and Behavioral Impacts of Alarms and Alerts Across the Spectrum of Diabetes Technology Use: a Cross-Sectional Study

**DOI:** 10.2196/97122

**Published:** 2026-08-13

**Authors:** Estelle Everett, Erin Shaw, Bryan Escobar Barrios, Beatrice Brumley, Kyrstin Lane, Tannaz Moin, Lauren E Wisk

**Affiliations:** 1 Division of Endocrinology, David Geffen School of Medicine, University of California, Los Angeles, 1100 Glendon Ave, Ste 850, Los Angeles, CA, 90024, United States, 1 310-486-6916; 2 Division of General Internal Medicine & Health Services Research, David Geffen School of Medicine, University of California, Los Angeles, Los Angeles, CA, United States; 3 VA Greater Los Angeles Healthcare System, Los Angeles, CA, United States; 4 HSR&D Center for the Study of Healthcare Innovation, Implementation, and Policy, VA Greater Los Angeles Healthcare System, Los Angeles, CA, United States; 5 Department of Health Policy and Management, Fielding School of Public Health, University of California, Los Angeles, Los Angeles, CA, United States

**Keywords:** insulin pump, continuous glucose monitoring, CGM, automated insulin delivery, alarm fatigue, diabetes distress, type 1 diabetes, quality of life

## Abstract

**Background:**

Continuous glucose monitors (CGMs), sensor-augmented pumps (SAPs), and automated insulin delivery (AID) systems have substantially improved glycemic outcomes for people with type 1 diabetes (T1D). However, these technologies also generate frequent alarms and alerts that may contribute to emotional burden, alarm fatigue, and maladaptive behavioral responses. Despite increasing recognition of alarm-related distress, little is known about how alarm burden differs across contemporary diabetes technologies.

**Objective:**

This study evaluated differences in alarm frequency, emotional burden, and behavioral responses to alarms and alerts among adults using CGMs alone, SAP therapy, and AID systems.

**Methods:**

We conducted a cross-sectional survey of adults (≥18 years) with T1D receiving care within a large academic health system between August 2024 and March 2025. Eligible participants completed an online survey assessing demographics, diabetes technology use, perceptions of alarm frequency and burden, and responses to alarms and alerts. Participants were categorized as CGM-only, SAP, or AID users. Differences between groups were evaluated using *χ*^2^ tests and ANOVA. Ordinal logistic regression models adjusted for age, gender, and diabetes duration were used to examine associations between diabetes technology type and alarm-related outcomes.

**Results:**

Among 838 respondents (mean age 46.0, SD 16.6 years; mean diabetes duration 23.0, SD 14.0 years; n=457, 55% women), AID users reported the highest alarm frequency, with 49% (n=252) experiencing alarms several times daily, compared with 35% (n=28) of SAP users and 32% (n=79) of CGM-only users (*P*<.001). AID users also reported greater alarm disruptiveness, annoyance, and unwanted attention than CGM-only users. Overcorrection of glucose levels in response to alerts was common across all technologies: 44% (n=371) and 50% (n=419) of participants reported sometimes overcorrecting low and high glucose levels, respectively, while 32% (n=265) and 18% (n=147) reported often or always overcorrecting low and high glucose levels, respectively. After adjustment, AID users had greater odds of frequent alarms (odds ratio [OR] 1.98, 95% CI 1.48-2.65), alarm disruptiveness (OR 1.67, 95% CI 1.24-2.25), annoyance (OR 1.65, 95% CI 1.23-2.19), unwanted attention (OR 1.85, 95% CI 1.38-2.47), ignoring alarms (OR 1.86, 95% CI 1.40-2.47), and overcorrecting low glucose levels (OR 1.61, 95% CI 1.20-2.15) compared with CGM-only users. Perceived effectiveness of alarms did not differ by technology type.

**Conclusions:**

Although AID systems provide important clinical benefits, they are associated with the greatest alarm and alert burden. Alarm-driven behaviors, including ignoring alerts and overcorrecting glucose levels, represent previously underrecognized consequences of diabetes technology that may diminish user experience and potentially affect glycemic management. These findings identify opportunities to improve alert algorithms while highlighting the need for individualized patient education, clinician engagement in setting appropriate alert thresholds, and routine assessment of alarm burden to optimize both glycemic outcomes and patient-centered experiences with diabetes technology.

## Introduction

Over the past 2 decades, advances in diabetes technology, particularly continuous glucose monitors (CGMs), insulin pumps, and automated insulin delivery (AID) systems, have transformed diabetes self-management. These innovations enable real-time glucose tracking, trend visualization, and algorithm-driven insulin adjustments, leading to improved glycemic outcomes and enhanced safety for many individuals with diabetes [1-5]. While diabetes technologies are clinically beneficial, they can also introduce substantial user burden [6]. One of the most pervasive sources of frustration stems from frequent device alarms and alerts. These notifications, intended to safeguard against hypo- and hyperglycemia, technical malfunctions, and connectivity issues, can be intrusive and exhausting.

Alarm and alert distress is increasingly recognized as an important contributor to the overall burden of diabetes technology use [6,7]. However, the emotional and behavioral impacts of alarms remain poorly understood. Studies assessing patient-reported outcomes have tended to use broad measures, such as the Diabetes Distress Scale, treatment satisfaction, or quality-of-life assessments, yielding inconsistent insights [6,8,9]. Few have specifically evaluated the frequency and impact of alarms and alerts, and those that have are predominantly qualitative [10-12] or limit their focus to a single technology type [13-16]. As a result, there is a significant gap in our understanding of how alarm burden differs across the full continuum of diabetes technologies, from CGM-only use to CGMs with an insulin pump in manual mode (sensor-augmented pump therapy; SAP) to the more advanced AID systems. Understanding how alarm frequency differs across diabetes technologies and how these alerts may influence emotional well-being and behavioral responses is critical for optimizing both device performance and patient experience and for facilitating shared decision-making around technology selection. The objective of this study was to compare the frequency and emotional burden of and behavioral responses to alarms and alerts among adults with type 1 diabetes (T1D) using a CGM only, SAP therapy, or an AID system. We hypothesized that increasing levels of technology automation would be associated with greater alarm burden and more frequent maladaptive behavioral responses.

## Methods

### Overview

We conducted a cross-sectional survey of adults (≥18 years) with T1D who used diabetes technology and received care within a large academic health system between August 2024 and March 2025. Eligible participants were identified using electronic medical record data and received invitations through their electronic patient portals. The invitation included an overview of the study and a link to an online survey administered using the Qualtrics online platform (Qualtrics LLC).

The survey included items assessing demographic (age, gender, race, ethnicity, education) and clinical characteristics (diabetes duration), current and prior use of diabetes technologies (including insulin pumps and CGMs), and perceptions of alarm and alert frequency, emotional burden, and response to alarms. Survey items were developed and refined through review of the existing literature, expert consensus, and consultation with adults living with T1D to ensure clarity and relevance. Questions were multiple choice, and responses were rated on 5-point Likert scales.

Participants were stratified by current level of diabetes technology use: CGM only, SAP, or AID systems. Participants who said they used the AID system on their pump every day or most days (5-7 days per week) were categorized as AID users; all other respondents with both a pump and a CGM were categorized as SAP users.

All analyses were conducted in SAS (version 9.4; SAS Institute Inc). All tests were 2-sided with an α criterion of .05. Demographic characteristics of the sample were compared by diabetes technology use, and appropriate bivariate tests were conducted (*χ*^2^ and ANOVA). We used *χ*^2^ tests to compare the prevalence of each of 9 questions about alarms and alerts by diabetes technology use. Differences in mean scores for alarm and alert questions were also compared by diabetes technology use and tested using ANOVA (Table S1). Ordinal logistic regression models were used to evaluate differences in alarm and alert scores by diabetes technology use, adjusting for age, diabetes duration, and gender (as these characteristics differed by technology use). For negatively framed outcomes, odds ratios (ORs) greater than 1.0 indicate increased odds of worse alarm and alert burden (eg, greater alert frequency, increased experience of alarm disruptiveness). For positively framed outcomes (alarm effectiveness and safety features), ORs greater than 1.0 indicate a greater endorsement of those attributes. The reporting of this cross-sectional study follows STROBE (Strengthening the Reporting of Observational Studies in Epidemiology) guidelines.

### Ethical Considerations

This study was reviewed and approved by the University of California, Los Angeles Institutional Review Board (FWA00004642) on March 4, 2024 (UCLA IRB-23‐1484). All participants provided informed consent prior to participation. A waiver of signed informed consent was granted in accordance with the Code of Federal Regulations [17]. Participants reviewed an institutional review board–approved study information statement prior to initiating the survey and provided electronic consent by selecting “yes” to participate. Participants who selected “no” were exited from the survey and no data were collected from them.

## Results

**Figure 1.**
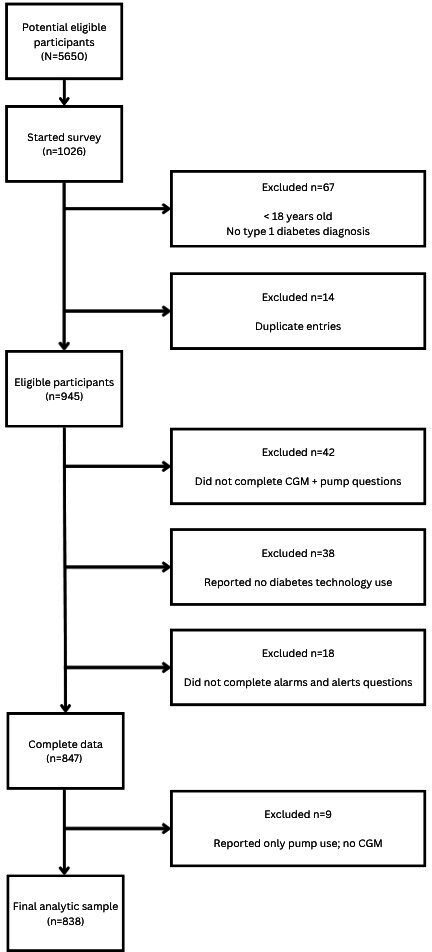
Flowchart of participants included in the final analytic sample. CGM: continuous glucose monitor.

We invited 5650 adults with T1D to participate in the survey between 2024 and 2025, and a total of 1026 (18%) responded. Of these, we excluded 67 participants who did not have T1D or were younger than 18 years of age, and we further excluded 14 duplicate records. Among the 945 remaining eligible participants, we excluded those who provided incomplete answers to the CGM and pump questions (n=42), reported no diabetes technology use (n=38), or provided incomplete answers to the alarm and alert questions (n=18). Due to the small number of insulin pump–only users (n=9), these participants were also excluded from the analysis. The final analytic sample comprised 838 eligible participants (Figure 1).

The characteristics of the participants are reported in Table 1. Overall, participants’ mean age was 45.76 (SD 16.91) years, and mean diabetes duration was 22.84 (SD 13.96) years. Mean age differed significantly across groups; AID users were younger (mean age 43.86, SD 16.03 years) than SAP users (mean age 50.13, SD 18.95 years) and CGM-only users (mean age 48.36, SD 17.47 years; *P*<.001). CGM-only and AID users had shorter mean diabetes durations of 20.11 (SD 15.01) years and 23.59 (SD 13.20) years, respectively, compared with SAP users (mean 26.39, SD 14.20 years; *P*<.001). Men were more likely to be CGM-only users (n=125, 51%; *P*<.001). There were no significant differences in type of technology used by race and ethnicity or educational attainment. Mean duration of CGM use was 5.10 (SD 4.20) years among CGM-only users and longer among SAP (mean duration 8.14, SD 5.58 years) and AID users (mean duration 7.99, SD 4.78 years; *P*<.001). Mean duration of insulin pump use did not differ significantly between SAP and AID users (12.42, SD 8.71 vs 12.52, SD 8.46 years; *P*=.92).

**Table 1.** Characteristics of participants.

Variables	All (n=838)	CGM^a^ only (n=244)	CGM and pump—manual mode (SAP^b^; n=79)	CGM and pump—automated mode (AID^c^; n=515)	*P* value
Age (years), mean (SD)	45.76 (16.91)	48.36 (17.47)	50.13 (18.95)	43.86 (16.03)	<.001
Age by subgroup (years), n (%)					<.001
18‐29	164 (20)	46 (19)	16 (20)	102 (20)	
30‐39	206 (25)	39 (16)	15 (19)	152 (29)	
40‐49	135 (16)	44 (18)	10 (13)	81 (16)	
50‐59	120 (14)	37 (15)	10 (13)	73 (14)	
60‐69	126 (15)	45 (18)	13 (16)	68 (13)	
≥70	87 (10)	33 (14)	15 (19)	39 (8)	
Diabetes duration (years), mean (SD)	22.84 (13.96)	20.11 (15.01)	26.39 (14.20)	23.59 (13.20)	<.001
Diabetes duration by subgroup (years), n (%)					<.001
0‐5	89 (11)	47 (19)	4 (5)	38 (7)	
6‐10	98 (12)	41 (17)	6 (8)	51 (10)	
11‐15	110 (13)	22 (9)	10 (13)	78 (15)	
16‐20	117 (14)	33 (14)	14 (18)	70 (14)	
21‐25	108 (13)	25 (10)	8 (10)	75 (15)	
26‐30	78 (9)	18 (7)	11 (13)	49 (9)	
≥31	238 (28)	58 (24)	26 (33)	154 (30)	
Gender, n (%)					<.001
Women	457 (55)	108 (44)	42 (53)	307 (60)	
Men	339 (40)	125 (51)	32 (41)	182 (35)	
Other/missing	42 (5)	11 (5)	5 (6)	26 (5)	
Race and ethnicity, n (%)					.61
Hispanic	108 (13)	32 (13)	5 (6)	71 (14)	
Non-White, non-Hispanic	99 (12)	31 (13)	12 (15)	56 (11)	
White, non-Hispanic	586 (70)	168 (69)	57 (73)	361 (70)	
Missing	45 (5)	13 (5)	5 (6)	27 (5)	
Education, n (%)					
High school diploma or GED^d^ or less	27 (3)	13 (5)	2 (3)	12 (2)	.35
Some college (no degree)	120 (14)	39 (16)	11 (14)	70 (14)	
Associate’s degree	55 (7)	19 (8)	5 (6)	31 (6)	
Bachelor’s degree	340 (41)	104 (42)	28 (35)	208 (40)	
Master’s degree	183 (22)	43 (18)	22 (28)	118 (23)	
PhD, MD, or other advanced degree	79 (9)	17 (7)	7 (9)	55 (11)	
Prefer not to say/missing	34 (4)	9 (4)	4 (5)	21 (4)	
Duration of CGM use (years), mean (SD)	7.16 (4.88)	5.10 (4.20)	8.14 (5.58)	7.99 (4.78)	<.001
Duration of CGM use by subgroup (years), n (%)					<.001
0‐5	367 (44)	158 (65)	29 (37)	180 (35)	
6‐10	335 (40)	68 (28)	35 (44)	232 (45)	
≥11	136 (16)	18 (7)	15 (19)	103 (20)	
Duration of pump use^e^ (years), mean (SD)	12.51(8.49)	—^f^	12.42 (8.71)	12.52 (8.46)	.92
Duration of pump use by subgroup (years), n (%)					.92
0‐5	167 (28)	—	23 (29)	144 (28)	
6‐10	114 (19)	—	18 (23)	96 (19)	
11‐15	108 (18)	—	9 (11)	99 (19)	
16‐20	90 (15)	—	15 (19)	75 (14)	
≥21	115 (20)	—	14 (18)	101 (20)	

a CGM: continuous glucose monitor.

b SAP: sensor-augmented pump.

c AID: automated insulin delivery.

d GED: General Education Development.

e Participants who use any type of pump, n=594.

f Not applicable.

The frequency of alarms and alerts differed significantly across technology groups (*P*<.001; Table 2). AID users reported the highest frequency, with 49% (n=252) experiencing alarms or alerts several times per day, compared with 35% (n=28) of SAP users and 32% (n=79) of CGM-only users. The psychological impact of these alarms was also greatest among insulin pump users: 32% (n=165) of AID and 30% (n=24) of SAP users reported that alarms and alerts were often or always disruptive compared with 21% (n=50) of CGM-only users (*P*=.009). Similarly, 41% (n=207) of AID users and 37% (n=29) of SAP users found alarms and alerts often or always annoying versus 28% (n=70) of CGM-only users. Alarms and alerts were also more likely to draw unwanted attention among pump users, with 21% (n=111) of AID and 24% (n=19) of SAP users reporting this often or always compared with 14% (n=34) of CGM-only users.

**Table 2.** Differences in alarm and alert (AA) frequency, burden, and responses by diabetes technology.

Variables	All (n=838), n (%)	CGM^a^ only (n=244), n (%)	CGM and pump—manual mode (SAP^b^; n=79), n (%)	CGM and pump—automated mode (AID^c^; n=515), n (%)	*P* value
AA frequency					<.001
Several times a day	359 (43)	79 (32)	28 (35)	252 (49)	
About once a day	246 (29)	67 (28)	21 (27)	158 (31)	
Every few days	156 (19)	65 (27)	18 (23)	73 (14)	
About once a week	49 (6)	18 (7)	3 (4)	28 (5)	
Never	28 (3)	15 (6)	9 (11)	4 (1)	
AA disruptiveness					.009
Never	36 (4)	16 (7)	6 (8)	14 (3)	
Rarely	163 (19)	56 (23)	18 (23)	89 (17)	
Sometimes	400 (48)	122 (49)	31 (39)	247 (48)	
Often	208 (25)	44 (18)	21 (26)	143 (28)	
Always	31 (4)	6 (3)	3 (4)	22 (4)	
AA unwanted attention					<.001
Never	97 (12)	47 (19)	10 (13)	40 (8)	
Rarely	247 (29)	78 (32)	26 (33)	143 (28)	
Sometimes	330 (39)	85 (35)	24 (30)	221 (43)	
Often	134 (16)	27 (11)	17 (21)	90 (17)	
Always	30 (4)	7 (3)	2 (3)	21 (4)	
AA annoyance					<.001
Never	60 (7)	28 (12)	10 (13)	22 (4)	
Rarely	152 (18)	52 (21)	12 (15)	88 (17)	
Sometimes	320 (38)	94 (39)	28 (35)	198 (38)	
Often	219 (26)	57 (23)	21 (27)	141 (28)	
Always	87 (11)	13 (5)	8 (10)	66 (13)	
AA effectiveness					.27
Never	14 (2)	7 (3)	1 (1)	6 (1)	
Rarely	79 (9)	22 (9)	7 (9)	50 (10)	
Sometimes	257 (31)	84 (34)	25 (32)	148 (29)	
Often	316 (38)	77 (32)	34 (43)	205 (40)	
Always	172 (20)	54 (22)	12 (15)	106 (20)	
AA safety feature					.03
Never	11 (1)	5 (2)	1 (1)	5 (1)	
Rarely	57 (7)	17 (7)	10 (13)	30 (6)	
Sometimes	176 (21)	36 (15)	13 (16)	127 (25)	
Often	255 (30)	76 (31)	22 (28)	157 (30)	
Always	339 (41)	110 (45)	33 (42)	196 (38)	
Overcorrecting highs					.21
Never	38 (4)	14 (6)	7 (9)	17 (3)	
Rarely	234 (28)	58 (24)	21 (26)	155 (30)	
Sometimes	419 (50)	123 (50)	41 (52)	255 (50)	
Often	140 (17)	47 (19)	10 (13)	83 (16)	
Always	7 (1)	2 (1)	0 (0)	5 (1)	
Overcorrecting lows					.03
Never	34 (4)	12 (5)	7 (9)	15 (3)	
Rarely	168 (20)	62 (26)	16 (20)	90 (17)	
Sometimes	371 (44)	106 (43)	30 (38)	235 (46)	
Often	216 (26)	54 (22)	20 (25)	142 (28)	
Always	49 (6)	10 (4)	6 (8)	33 (6)	
Ignoring AA					<.001
Never	111 (13)	40 (17)	18 (23)	53 (10)	
Rarely	229 (27)	86 (35)	14 (18)	129 (25)	
Sometimes	240 (29)	69 (28)	25 (32)	146 (28)	
Often	218 (26)	42 (17)	17 (21)	159 (31)	
Always	40 (5)	7 (3)	5 (6)	28 (6)	

a CGM: continuous glucose monitor.

b SAP: sensor-augmented pump.

c AID: automated insulin delivery.

Responses to alarms and alerts differed across technology groups. Over one-third of AID users (n=258, 37%) reported often or always ignoring alarms compared with 27% (n=22) of SAP users and 20% (n=49) of CGM-only users (*P*<.001). Approximately one-third of both AID (n=175, 34%) and SAP (n=26, 33%) users reported often or always overcorrecting low glucose levels by dosing too little insulin or treating low blood glucose with food instead of adjusting insulin in response to alerts compared with 26% (n=64) of CGM-only users (*P*=.03). In contrast, overcorrection of high glucose levels often or always by dosing too much insulin in response to alerts was reported more frequently among CGM-only users (n=49, 20%) than among AID (n=88, 17%) and SAP users (n=10, 13%).

Despite these differences in behavioral responses to alarms, most participants found alarms and alerts to be effective, with 60% (n=311) of AID, 58% (n=46) of SAP, and 54% (n=131) of CGM-only users reporting that alarms and alerts were often or always helpful (*P*=.27). However, CGM-only users were more likely to view alarms and alerts as an important safety feature (76%) compared with SAP (70%) and AID users (68%; *P*=.03).

In adjusted models (Table 3), AID users reported a higher frequency of alarms and alerts compared with CGM-only users (OR 1.98, 95% CI 1.48‐2.65), as well as greater perceived disruptiveness (OR 1.67, 95% CI 1.24‐2.25), annoyance (OR 1.65, 95% CI 1.23‐2.19), and unwanted attention (OR 1.85, 95% CI 1.38‐2.47). There were no significant differences in perceived effectiveness (OR 0.80, 95% CI 0.60‐1.07) or in viewing alarms and alerts as an important safety feature (OR 1.31, 95% CI 0.98‐1.76). AID users were also more likely to ignore alarms (OR 1.86, 95% CI 1.40‐2.47) and overcorrect low glucose levels (OR 1.61, 95% CI 1.20‐2.15). No differences were observed in overcorrection of high glucose levels (OR 0.85, 95% CI 0.63‐1.15).

**Table 3.** Adjusted associations between diabetes technology type and alarm and alert (AA) frequency, burden, and responses.

Variables	AA frequency, OR^a^ (95% CI)	AA disruptiveness, OR (95% CI)	AA unwanted attention, OR (95% CI)	AA annoyance, OR (95% CI)	AA effectiveness, OR (95% CI)	AA safety feature, OR (95% CI)	Overcorrecting highs, OR (95% CI)	Overcorrecting lows, OR (95% CI)	Ignoring AAs, OR (95% CI)
Device/tech use									
SAP^b^ vs CGM^c^	1.03 (0.65‐1.64)	1.27 (0.79‐2.05)	1.65 (1.03‐2.64)	1.35 (0.85‐2.15)	0.97 (0.61-1.55)	1.37 (0.86-2.20)	0.69 (0.42-1.11)	1.24 (0.78-1.99)	1.45 (0.91-2.29)
AID^d^ vs CGM	1.98 (1.48‐2.65)	1.67 (1.24‐2.25)	1.85 (1.38‐2.47)	1.65 (1.23-2.19)	0.80 (0.60-1.07)	1.31 (0.98-1.76)	0.85 (0.63-1.15)	1.61 (1.20-2.15)	1.86 (1.40-2.47)
Age	0.98 (0.97‐0.99)	0.99 (0.99‐1.00)	0.99 (0.98‐1.00)	0.98 (0.97-0.99)	0.99 (0.98-0.99)	0.98 (0.98-0.99)	0.99 (0.98-1.00)	1.00 (0.99-1.01)	0.97 (0.96-0.98)
Diabetes duration	1.01 (1.00‐1.02)	0.99 (0.99‐1.00)	0.99 (0.98‐1.00)	1.00 (0.99-1.01)	1.02 (1.01-1.03)	1.00 (0.99-1.01)	1.01 (1.00-1.02)	0.99 (0.98-1.00)	1.00 (0.99-1.01)
Gender									
Men vs women	0.92 (0.71‐1.20)	0.69 (0.53‐0.90)	0.69 (0.53‐0.89)	0.67 (0.52-0.87)	1.13 (0.87-1.46)	1.50 (1.16-1.96)	1.35 (1.03-1.77)	1.06 (0.81-1.38)	1.08 (0.84-1.40)
Other vs women	1.08 (0.60‐1.95)	1.17 (0.65‐2.10)	0.74 (0.42‐1.33)	1.44 (0.81-2.56)	0.66 (0.37-1.18)	0.88 (0.49-1.59)	1.29 (0.71-2.34)	1.34 (0.75-2.39)	1.29 (0.73-2.29)

a OR: odds ratio.

b SAP: sensor-augmented pump.

c CGM: continuous glucose monitor.

d AID: automated insulin delivery.

In adjusted models comparing SAP to CGM-only users, SAP users reported greater feelings of unwanted attention (OR 1.65, 95% CI 1.03‐2.64) but did not differ significantly in alarm frequency, disruptiveness, annoyance, perceived effectiveness, safety perception, or responses to alarms (ignoring or overcorrecting).

Alarm responses varied by demographic characteristics. Although there were no gender differences in reported alarm frequency (OR 0.92, 95% CI 0.71‐1.20), men were less likely than women to perceive alarms as disruptive (OR 0.69, 95% CI 0.53‐0.90) or to report that alarms drew unwanted attention (OR 0.69, 95% CI 0.53‐0.89). Men were more likely to view alarms as an important safety feature (OR 1.50, 95% CI 1.16‐1.96) and were more likely to overcorrect high glucose levels in response to alerts (OR 1.35, 95% CI 1.03‐1.77).

## Discussion

In this cross-sectional study of adults with T1D using the spectrum of contemporary diabetes technologies, we compared alarm frequency, emotional burden, and behavioral responses across CGM-only, SAP, and AID system users. We found that AID users experienced the greatest alarm burden, reporting more frequent and disruptive alarms and greater maladaptive behavioral responses to alarms than users of less automated technologies. These findings highlight an important trade-off between the clinical benefits of automation and the psychosocial burden associated with increasing device complexity.

Several factors likely contribute to the greater alarm burden observed among AID users. The combination of insulin pump and CGM use may increase alarm burden because each device can generate alerts through separate safety and monitoring mechanisms. Common causes of insulin pump alerts include obstruction of insulin flow, infusion set or site problems, and device or reservoir issues [11,18]. Additionally, a CGM may issue alerts due to true glycemic excursions, but such alerts can also result from sensor inaccuracies, compression lows (eg, due to sleep position), loss of connectivity, or device malfunction [19,20].

The heightened alarm burden among AID users likely reflects both the integrated nature of these systems and their continuous feedback mechanisms. AID systems include several unique alarms and alerts tied to their control algorithms and safety features, including alerts when the algorithm cannot safely determine insulin delivery, mode transition alarms when switching between automated and manual operation, and notifications for automated correction boluses. AID systems also issue predictive safety alarms for impending hypo- or hyperglycemia and alerts for potential system failures or overrides. In addition, many contemporary insulin pumps include integrated speakers and can generate audible glucose-level alerts based on connected CGM data, while the same glucose event may simultaneously trigger alerts on a smartphone, dedicated receiver, or smartwatch. Consequently, users may receive multiple alerts for a single glucose excursion, each requiring individual acknowledgment or dismissal, further amplifying alarm burden. While these alerts are critical for safety and algorithm performance, the sheer frequency may contribute to alarm fatigue and reduced responsiveness over time. Our finding that AID users were more likely to ignore alarms underscores this concern and parallels alarm fatigue phenomena reported in other health care settings, such as bedside monitoring [21-23].

Our findings also suggest that frequent alarms and alerts may lead to maladaptive behavioral responses, including overcorrection in response to both low and high glucose–level alerts. Across our cohort of 838 respondents, 44% (n=371) and 50% (n=419) of participants reported sometimes overcorrecting for low and high glucose levels, respectively, and 32% (n=265) and 18% (n=147) reported often or always overcorrecting for low and high glucose levels, respectively, in response to alerts. This represents a novel phenomenon that has not been well characterized in prior diabetes technology research. These behaviors may reflect heightened vigilance or anxiety triggered by frequent alerts, prompting users to respond preemptively or excessively to perceived glucose excursions. While glycemic alarms are intended to promote safety, repeated overcorrection may paradoxically increase glucose variability and contribute to a “roller-coaster” pattern of glycemia, with rapid swings from high to low or vice versa. Further investigation is warranted to quantify how often overcorrection precipitates such fluctuations and to understand its downstream impact on key glycemic outcomes, including hemoglobin A_1c_ level, time in range, and hypoglycemia risk, particularly among users of AID systems.

Psychological and behavioral responses to alarms varied by gender. Women reported higher levels of disruptiveness and unwanted attention, consistent with prior research showing that female [10,24-27] users may experience greater diabetes-related burden and social visibility concerns. Men were more likely to view alarms as a safety feature and more likely to overcorrect highs, which may reflect differences in risk perception and response behaviors by gender [28,29]. These patterns highlight the need for tailored education and support that address demographic and psychosocial differences in alarm experiences.

Our study adds to the growing literature emphasizing the importance of user-centered outcomes in diabetes technology evaluation. While glycemic metrics remain the gold standard for assessing device efficacy, psychosocial factors, including alarm fatigue, distress, and technology satisfaction, strongly influence sustained device use and quality of life. Our findings suggest that alarm burden should be considered alongside glycemic outcomes when selecting, initiating, and optimizing diabetes technologies. Although AID and SAP users experienced greater alarm-related distress, perceived effectiveness and safety of alarms were similar across device types, suggesting that users recognize the clinical value of alerts despite their inconvenience. This coexistence of perceived utility and burden highlights a central design challenge: reducing unnecessary alerts while preserving safety-critical notifications. Clinicians should routinely assess alarm-related distress during diabetes visits and work with patients to personalize alarm settings, optimize notification thresholds, and address maladaptive responses such as alarm avoidance and overcorrection. These findings also underscore the importance of ongoing patient education to help individuals understand the purpose of alerts, develop appropriate responses, and safely manage frequent notifications without disengaging from their devices. Patients experiencing frequent alarms may benefit from periodic reassessment of alert settings and additional clinical support to ensure that alarms remain both meaningful and actionable. Likewise, manufacturers should continue to refine algorithms, personalize notification thresholds, and apply user-centered design principles to develop alert systems that are responsive, flexible, and minimally intrusive.

The limitations of this study include its cross-sectional design, which precludes causal inference. Participants were drawn from a single academic health system, which may limit generalizability. Self-reported measures may also be subject to recall or response bias. Additionally, we used a study-specific survey rather than a standardized, validated patient-reported outcome measure. While we assessed perceived burden and behavioral responses, we did not directly measure alarm frequency or glycemic data using device downloads. We also did not evaluate differences in alarm burden across individual CGM or insulin pump systems; however, device-specific features, including differences in alert modalities, may influence users’ experiences and should be examined in future studies.

This study also has several strengths, including its large sample, literature-informed survey instrument, and inclusion of users across the full spectrum of contemporary diabetes technologies. To our knowledge, this is the first study to quantitatively compare alarm frequency, burden, and behavioral responses across these technologies. Future studies incorporating objective device data and longitudinal follow-up will be important to determine how alarm burden influences glycemic outcomes and long-term technology engagement.

In conclusion, as diabetes technologies become increasingly automated, optimizing the user experience must become a priority alongside improving glycemic outcomes. Addressing alarm-related distress and its psychological and behavioral consequences is essential to ensure that advances in automation translate into meaningful improvements in quality of life, sustained technology engagement, and clinical outcomes. A user-centered approach to device design, individualized patient education, and proactive clinical management, including routine review of alarm settings and patient responses to alerts, will be critical to realizing the full potential of diabetes technologies.

## Abbreviations

AID: automated insulin delivery
CGM: continuous glucose monitor
OR: odds ratio
SAP: sensor-augmented pump
STROBE: Strengthening the Reporting of Observational Studies in Epidemiology
T1D: type 1 diabetes
